# Pediatric Esophageal Biomechanics and Remodeling: Integrating Distensibility and Compliance Beyond Luminal Diameter

**DOI:** 10.1111/jgh.70570

**Published:** 2026-07-13

**Authors:** Brett J. Hoskins, Kenneth Ng, Michael A. Manfredi

**Affiliations:** ^1^ Division of Pediatric Gastroenterology, Hepatology, and Nutrition, Department of Pediatrics Indiana University School of Medicine, Riley Hospital for Children at IU Health Indianapolis Indiana USA; ^2^ Division of Pediatric Gastroenterology, Hepatology, and Nutrition, Department of Pediatrics The Johns Hopkins University School of Medicine Baltimore Maryland USA; ^3^ Division of Gastroenterology, Hepatology and Nutrition, Department of Pediatrics Children's Hospital of Philadelphia Philadelphia Pennsylvania USA

**Keywords:** child, endoscopy, eosinophilic esophagitis, esophageal diseases, esophageal motility disorders, gastrointestinal

## Abstract

Management of pediatric esophageal disease has traditionally relied on static anatomic endpoints including luminal diameter, endoscopic findings, and histologic features to guide diagnosis and therapeutic decision‐making. However, these measures incompletely capture esophageal function and the biomechanical consequences of chronic inflammation, fibrosis, injury, and extrinsic compression. In adult gastroenterology, esophageal distensibility assessment using the functional lumen imaging probe (FLIP) has emerged as an adjunctive marker of disease severity and treatment response in conditions such as achalasia and eosinophilic esophagitis.

This work reviews pediatric and adult literature examining esophageal distensibility, compliance, and biomechanical remodeling, with emphasis on FLIP‐based assessment, disease‐specific remodeling patterns, and implications for therapeutic endoscopy. Available evidence demonstrates that esophageal distensibility provides physiologic information beyond static luminal diameter and may help explain discordance between anatomic findings and symptoms. Pediatric studies support the feasibility of FLIP‐based functional assessment and suggest that altered distensibility trajectories reflect disease‐related remodeling in eosinophilic esophagitis, caustic injury, postsurgical strictures, and vascular compression. Functional measurements may also help interpret dilation response and identify situations in which additional size escalation alone may yield diminishing functional benefit. This review also proposes a pediatric framework integrating FLIP‐derived biomechanics into interpretation of dysphagia, dilation response, and therapeutic decision‐making.

Esophageal distensibility and biomechanical remodeling represent underutilized actionable concepts in pediatric esophageal disease. Integrating functional assessment with anatomic evaluation may improve interpretation of disease severity, explain discordance between symptoms and luminal diameter, and inform therapeutic decision‐making. Pediatric‐specific interpretive frameworks are needed to determine how distensibility and compliance metrics can guide management in children.

## Graphical abstract

Esophageal distensibility and compliance provide physiologic information beyond luminal diameter alone in pediatric esophageal disease. This review proposes a practical biomechanics‐informed framework integrating FLIP assessment into interpretation of dysphagia, esophageal remodeling, dilation response, and therapeutic decision‐making in children.

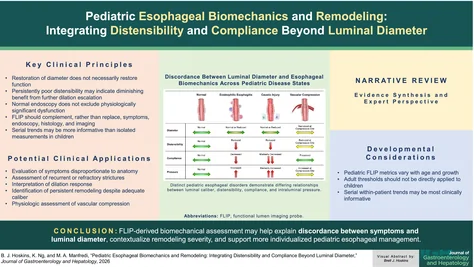

## 1 Introduction

Pediatric esophageal diseases encompass a diverse group of conditions characterized by inflammation, fibrosis, structural narrowing, extrinsic compression, or primary motility disorders. Across these disease states, clinical management has historically centered on anatomic assessment, most commonly luminal diameter measured endoscopically or radiographically—and symptom response following intervention. Although practical, this approach assumes that restoration of luminal caliber equates to restoration of function [1, 2].

Clinical experience increasingly challenges this assumption. Children may continue to experience dysphagia or other feeding‐related symptoms despite achieving “acceptable” luminal diameter after dilation. Conversely, some children tolerate relatively narrow lumens with minimal symptoms. These discrepancies illustrate a key limitation of diameter‐based assessment: Static anatomy incompletely captures esophageal function [1, 3].

In adult practice, attention has shifted toward esophageal distensibility as an adjunctive functional measure of esophageal wall behavior under physiologic stress, with growing interest in compliance as a complementary biomechanical metric [3, 4, 5, 6]. These concepts are particularly relevant in pediatrics, where growth, tissue plasticity, and prolonged disease trajectories amplify the consequences of chronic remodeling. Despite this, pediatric‐specific data on esophageal biomechanics, including compliance, remain limited.

This review explores the concept that esophageal biomechanics, including both distensibility and compliance, along with remodeling, should be considered important components of pediatric esophageal disease assessment. Rather than reframing compliance as an alternative measurement to luminal diameter, we propose it as a complementary functional endpoint that contextualizes anatomic findings and better reflects disease biology. By broadening how disease severity and treatment success are defined, a biomechanics‐informed framework offers opportunities to better guide therapeutic decision‐making, tailor interventions, and refine longitudinal outcomes in children. Importantly, the goal of this review is not to define rigid pediatric thresholds but to outline how functional metrics can be integrated into clinical reasoning and therapeutic decision‐making in a developmentally dynamic population.

## 2 Conceptualizing Esophageal Compliance and Remodeling in Children

### 2.1 Definitions

Esophageal compliance refers to the ability of the esophageal wall to distend in response to applied pressure. Distensibility is a related metric, often expressed as luminal cross‐sectional area or derived diameter relative to intraluminal pressure. Remodeling describes structural and biomechanical changes in the esophageal wall over time, typically resulting from chronic inflammation, fibrosis, ischemia, or mechanical stress [5, 7].

While these terms are well established in adult literature, their application in pediatrics requires special consideration. The pediatric esophagus is not a static structure; it undergoes ongoing growth and developmental remodeling throughout childhood and adolescence. As such, compliance must be interpreted relative to age, size, and developmental stage [1].

Distensibility and compliance, while related, capture distinct biomechanical properties of the esophageal wall. Distensibility is typically expressed as cross‐sectional area (or diameter) relative to intraluminal pressure at a standardized distension and reflects a static geometric endpoint. Compliance, in contrast, describes the dynamic relationship between volume and pressure (Δvolume/Δpressure) across sequential distension steps and reflects the tissue's capacity to accommodate incremental load [5, 7].

Adult studies using the functional lumen imaging probe (FLIP; EndoFLIP, Medtronic, Minneapolis, MN, USA) demonstrate clinically meaningful discordance between distensibility and compliance metrics. This balloon‐based impedance planimetry system measures luminal cross‐sectional area and intrabag pressure during controlled distension. Some patients exhibit reduced compliance despite a preserved distensibility plateau, or vice versa, highlighting the limitations of diameter‐based assessment alone [5]. Pediatric FLIP studies suggest that compliance metrics may capture early or diffuse biomechanical alterations that are not reflected by distensibility alone, particularly in eosinophilic esophagitis [3]. In current adult clinical practice, distensibility metrics derived from FLIP are used more commonly than compliance to guide therapeutic decisions, whereas compliance measurements remain primarily investigational and are not routinely incorporated into standard FLIP reporting [5, 6, 7].

Across injury etiologies, esophageal remodeling generally follows a phased wound‐healing trajectory, progressing from acute inflammation and tissue injury to collagen deposition and eventual fibrosis [8, 9]. The resulting increase in wall stiffness may alter pressure–volume behavior and manifest as either focal stricture formation or diffuse biomechanical stiffening without critical luminal narrowing [10].

### 2.2 Diameter Is Insufficient to Predict Residual Symptomatology

**FIGURE 1:**
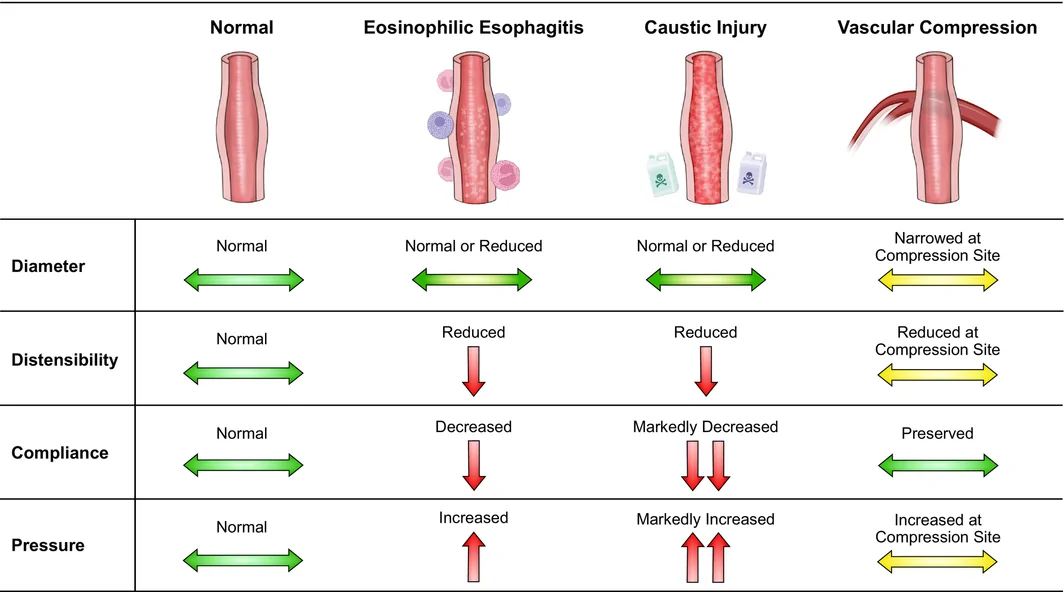
Discordance between luminal diameter and esophageal biomechanics across pediatric disease states. Conceptual schematic illustrating how anatomic luminal diameter may not fully reflect esophageal biomechanical function. Relationships between luminal diameter, distensibility, compliance, and intraluminal pressure are shown across representative pediatric disease states, including normal esophagus, eosinophilic esophagitis, caustic injury, and vascular compression. Arrow direction and color indicate relative changes in each parameter compared with normal. Green arrows indicate normal or preserved parameters, yellow arrows indicate variable or localized change, and red arrows indicate reduced compliance/distensibility or increased intraluminal pressure. Image credit: BioRender. Hoskins BJ. (2026). https://biorender.com/mdh87ar.

Luminal diameter, the primary geometric parameter used to guide dilation during endoscopy, provides a single static measurement that does not account for wall stiffness, elasticity, or biomechanical reserve. Adult studies using impedance planimetry demonstrate that distensibility and compliance metrics may diverge despite similar luminal diameters or cross‐sectional areas, reflecting differences in underlying pressure–volume relationships and tissue remodeling [5, 11]. These biomechanical discordances suggest that static geometric measurements alone incompletely characterize esophageal function (Figure 1). Similar paradigm shifts have occurred in other areas of medicine, where physiologic pressure–flow relationships have replaced static anatomic measurements for determining clinically meaningful obstruction; for example, cardiology now relies on fractional flow reserve to define the functional significance of coronary stenoses rather than angiographic diameter alone [12, 13].

In pediatrics, interpretation of luminal caliber is influenced by measurement methodology. Historically, visual comparative estimation using biopsy forceps demonstrated good concordance with objective measurements, particularly in smaller caliber esophagi, although precision decreased at larger diameters [2]. Even when diameter is accurately estimated, however, static measures do not capture dynamic wall behavior during distension, which may contribute to discordance between such measurements in relation to symptom burden and response to interventions [5, 11].

These limitations are particularly relevant in children, as esophageal distensibility appears to vary with age and fixed diameter thresholds do not account for developmental changes in esophageal biomechanics. Pediatric studies demonstrate that this expected age‐dependent trajectory is disrupted in eosinophilic esophagitis, reinforcing the pronounced limitations of static diameter‐based assessment across childhood [1].

## 3 Functional Assessment of the Pediatric Esophagus

### 3.1 Principles of Functional Lumen Assessment

Functional lumen imaging technologies measure esophageal geometry under controlled distension, providing real‐time data on diameter, cross‐sectional area, pressure, compliance, and distensibility. These measurements characterize the biomechanical behavior of the esophageal wall rather than its resting appearance and address known limitations of static diameter assessment during endoscopy [2].

Beyond balloon‐based approaches, adult studies using high‐resolution impedance manometry demonstrate that pressure–geometry relationships can also be assessed during physiologic swallowing, reinforcing that esophageal opening is phase‐dependent and context‐specific [4]. Together, these data support a broader biomechanical framework in which distension behavior reflects an integrated pressure–geometry relationship rather than a single diameter measurement. Within pediatric practice, high‐resolution manometry may be challenging in some patients, particularly young children or those with developmental delay who may be unable to tolerate awake catheter‐based physiologic testing. In these settings, functional assessment during sedated endoscopy becomes particularly valuable.

FLIP provides real‐time measurement of luminal cross‐sectional area and intrabag pressure during graded distension, enabling calculation of the distensibility index and characterization of contractile patterns (FLIP panometry) [7, 14]. In adult practice, clinical guidance supports FLIP as an adjunctive test when symptoms suggest obstruction despite high‐quality endoscopy and other physiologic testing [6, 15]. FLIP has also been applied in procedural settings, including assessment of esophagogastric junction opening during and after myotomy, as well as complementary intraoperative evaluation during anti‐reflux surgery [6].

### 3.2 Application in Pediatrics

In the pediatric population, functional assessment has been applied to conditions such as eosinophilic esophagitis, congenital and acquired strictures, postsurgical narrowing, and extrinsic vascular compression. Several pediatric studies demonstrate feasibility and safety, even in young children, though interpretation remains challenging due to the lack of established pediatric normative data [1, 3, 14].

Although the pediatric experience with FLIP now spans both diagnostic and therapeutic applications, age‐ and size‐adjusted reference values remain limited, necessitating cautious interpretation and reliance on physiologic trends rather than fixed thresholds. Pediatric studies support the feasibility and safety of FLIP during sedated endoscopy, including cohorts under 5 years of age, without increased procedure time or procedure‐related complications [14].

### 3.3 Protocol Standardization and Interpretive Frameworks

Recent adult expert guidance has proposed standardized FLIP panometry acquisition protocols, including sequential fill volumes with defined dwell times and categorical interpretation of esophagogastric junction opening based on distensibility index and maximal diameter [6]. FLIP panometry refers to the integrated assessment of luminal opening dynamics and contractile patterns during controlled balloon distension. Although developed in adult populations, these recommendations provide an important methodological scaffold for pediatric adaptation. Importantly, direct application of adult thresholds to children is inappropriate given differences in esophageal length, caliber, growth, and sedation effects.

A standardized approach to FLIP panometry performance and interpretation has recently been proposed by the Dallas Consensus, including protocol elements such as graded stepwise balloon distension, predefined dwell times, standardized reporting of distensibility index and maximal diameter, and a classification framework for contractile response patterns during sedation, along with updated terminology for motility patterns assessed during sedated endoscopy [16]. These recommendations provide a strong methodological foundation for pediatric practice; however, pediatric adoption requires age‐ and size‐aware interpretation. In practice, shorter FLIP catheter configurations (e.g., 8‐cm measurement segments) may be preferred in smaller children to accommodate esophageal length, whereas longer configurations (e.g., 16‐cm segments) are more feasible in larger children and adolescents. Balloon fill volumes and distensibility values should therefore be interpreted in relation to body size and esophageal length rather than absolute adult thresholds.

### 3.4 Limitations and Practical Considerations

Challenges include device size compatibility, sedation requirements, and the absence of robust pediatric normative ranges. Additionally, functional data must be interpreted within the broader clinical context, as compliance or distensibility alone does not define disease severity or treatment necessity.

Interpretation in children must account for developmental scaling, as pediatric esophageal length and size influence baseline distensibility, pressure–area relationships, and contractile patterns during distension. In healthy children, esophageal distensibility increases with age, reflecting growth‐related changes in esophageal length and caliber. Shorter esophageal length may affect measurement positioning in younger children. Accordingly, FLIP metrics should be interpreted relative to body size and growth rather than fixed adult thresholds, as applying adult‐derived cutoffs without adjustment risks misclassification [1, 17].

## 4 Disease‐Specific Patterns of Esophageal Remodeling

### 4.1 Eosinophilic Esophagitis

Chronic eosinophilic inflammation leads to subepithelial fibrosis and progressive stiffening of the esophageal wall. In children, symptoms may precede overt narrowing, making biomechanical assessment, including compliance, potentially valuable for early detection of fibrostenotic disease.

Contemporary EoE guidance emphasizes that symptoms, endoscopic appearance, and mucosal histology do not always capture fibrostenotic burden; functional tools such as impedance planimetry can provide objective assessment of luminal caliber and wall mechanics that complements standard evaluation in selected patients [18].

FLIP has clarified that esophageal remodeling in pediatric EoE is not fully captured by luminal narrowing. As noted above, early pediatric studies demonstrated that reduced esophageal compliance correlates with epithelial and subepithelial remodeling, even in the absence of critical diameter reduction [3]. Subsequent work showed that distensibility normally increases with age in healthy children but remains blunted in pediatric EoE, suggesting that chronic inflammation disrupts expected developmental biomechanics [1]. More recent studies have further refined this paradigm by demonstrating that reduced distensibility identifies children at higher risk for fibrostenotic complications, whereas longitudinal data indicate that histologic response is associated with improvement in distensibility and symptom burden [10, 19]. Notably, a subset of children demonstrates persistently abnormal mechanical properties despite histologic improvement, highlighting the potential role of compliance as a marker of residual or subclinical remodeling or incomplete therapeutic response [20].

In children and adolescents with EoE, DI derived from EndoFLIP correlates with histologic features of remodeling; DI at 30‐mL inflation demonstrates a negative correlation with composite EoE Histology Scoring System (EoEHSS) scores, supporting functional assessment as a complementary remodeling readout alongside biopsies [21].

Emerging work in adult EoE proposes physiomechanical classifications derived from FLIP panometry metrics (opening dynamics plus contractile response) that map to symptom duration and fibrostenotic endoscopic features, suggesting a path toward biomechanics‐informed severity staging [11]. Composite indices such as the C2D2 score—an adult‐derived FLIP panometry classification that integrates contractile response patterns and distensibility measures to stratify fibrostenotic severity—may also provide objective outcome measures for trials and longitudinal monitoring [22].

### 4.2 Caustic and Button Battery–Related Injury

Caustic and button battery–related injuries often result in circumferential necrosis followed by dense fibrosis. Luminal diameter may improve with dilation, yet compliance frequently remains impaired, predisposing patients to recurrent symptoms and repeat interventions.

Caustic ingestions initiate remodeling through depth‐dependent chemical injury. Strong alkalis can penetrate deeply via liquefactive necrosis with transmural injury extending into the muscularis propria, whereas acids more commonly cause superficial coagulative necrosis with eschar formation that may limit deeper penetration [8, 9, 23]. Progression from acute injury to collagen deposition and fibrosis results in circumferential scarring and persistent loss of compliance, particularly when injury extends beyond the mucosa [8, 9]. Endoscopic severity predicts downstream fibrostenosis; pediatric data support that higher injury grades (including Zargar grade 2B/3A patterns) are associated with later stricture development [24]. From a biomechanics standpoint, these strictures represent transmural fibrosis with persistent loss of compliance, explaining why clinical “success” defined purely by lumen caliber may not translate into durable symptom resolution.

Even when dilation restores luminal diameter, the cellular and extracellular matrix changes that drive stiffness may not be reversed. Older pediatric series demonstrate that caustic strictures often require a greater number of dilation sessions than other benign etiologies (mean 7.8 vs. 1.9 sessions), consistent with persistent remodeling despite anatomic gains [25]. This phenotype aligns with a compliance‐based framework: Dilation can increase caliber, but low compliance may persist and predict symptom recurrence, need for repeat intervention, and prolonged clinical trajectories. In addition, injury‐related impairment of peristalsis may contribute to persistent dysphagia, as dilation restores luminal caliber but does not correct underlying neuromuscular dysfunction [26, 27].

Button battery–related remodeling follows a distinct and accelerated mechanism. Tissue injury occurs through simultaneous electrochemical generation of hydroxide at the negative pole (driving rapid liquefactive necrosis), pressure necrosis at the impaction site, and potential leakage of alkaline contents [28, 29]. Severe damage can occur within hours, and the resulting circumferential full‐thickness injury can heal with aggressive fibrosis and early stenosis. Contemporary pediatric experiences and systematic reviews highlight that esophageal strictures are among the most frequent serious late sequelae after esophageal impaction, with complication risk influenced by dwell time and battery size, among other factors [30, 31]. For a compliance‐based paradigm, button battery injury is an archetypal model in which a mechanically stiff segment may persist despite acceptable lumen caliber, supporting functional assessment to guide longitudinal management.

### 4.3 Postsurgical Strictures

Anastomotic and postoperative strictures may exhibit heterogeneous remodeling, with focal stiffness that is poorly captured by diameter alone. Functional assessment can help distinguish fixed fibrotic narrowing from functionally adaptable segments.

Postsurgical strictures, particularly after esophageal atresia repair, represent a remodeling phenotype driven less by mucosal inflammation and more by focal ischemic and tension‐related fibrosis at the anastomosis. Mechanisms include anastomotic tension (especially in long‐gap repairs), local ischemia from surgical manipulation and suture placement, anastomotic leak with fibrotic healing, and chronic reflux‐mediated inflammation that perpetuates remodeling at the repair site [32, 33]. This helps explain a common clinical paradox: Mucosa may appear relatively normal endoscopically, whereas the submucosa and muscularis are mechanically rigid, producing a fixed, non‐distensible segment where diameter alone incompletely reflects swallow function. Recurrent or refractory strictures likely reflect an “aggressive remodeling” subtype with ongoing collagen deposition and high relapse rates, often prompting use of adjunctive approaches (e.g., intralesional steroids and topical mitomycin C) in addition to serial dilation [33, 34]. Moreover, children with repaired esophageal atresia frequently demonstrate abnormal motility patterns on high‐resolution manometry, reflecting neuromuscular dysfunction that may coexist with focal mechanical narrowing [35]. These findings further illustrate how functional metrics can reveal physiologic abnormalities that are not apparent from diameter‐based assessment alone. In this context, compliance‐based assessment may better match the underlying biology than static caliber thresholds.

### 4.4 Vascular Compression Syndromes

Extrinsic compression presents a unique paradigm in which luminal narrowing may fluctuate. Compliance assessment can clarify the functional significance of compression and guide decisions regarding intervention versus observation.

Vascular compression syndromes (vascular rings/slings) differ fundamentally from intrinsic strictures because the primary limitation is external constraint rather than primary wall fibrosis. Early in the disease course, intrinsic esophageal wall structure may be relatively preserved, whereas symptoms arise from variable narrowing that can fluctuate with respiration, cardiac cycle, bolus consistency, and distension demands [36, 37, 38]. Over time, chronic stasis and repeated mechanical compression may contribute to secondary changes (e.g., mucosal irritation or hypertrophy), but the key clinical distinction is whether symptoms reflect a “pure external clamp” versus superimposed intrinsic remodeling.

Diagnosis typically requires integration of symptom pattern with dynamic imaging—esophagram plus cross‐sectional angiographic imaging (CT angiography or MR angiography) to define anatomy and guide surgical planning—with bronchoscopy often performed as part of a multidisciplinary aerodigestive evaluation to assess associated airway pathology such as tracheomalacia [37, 38]. In a compliance‐based framework, impedance planimetry may help phenotype these patients by demonstrating preserved intrinsic distension behavior limited by external constraint versus a mechanically stiff segment suggestive of secondary remodeling.

Recent pediatric data suggested that FLIP may provide clinically meaningful complementary physiologic information in children with vascular esophageal compression. In a recent pediatric case series, FLIP identified functionally significant narrowing in 75% of children with vascular compression, including patients with normal endoscopic findings, and revealed discordance between anatomic imaging and physiologic obstruction. Paired measurements in two patients demonstrated significantly reduced diameter and distensibility at the compression site compared with the lower esophageal sphincter, supporting physiologic relevance beyond anatomic indentation alone [39].

Importantly, FLIP also helped exclude clinically significant functional obstruction in symptomatic children whose symptoms improved with non‐surgical management, highlighting its potential role in avoiding unnecessary surgical intervention [39]. Collectively, these findings support a biomechanics‐based distinction between pure extrinsic constraint with preserved intrinsic wall mechanics and secondary remodeling characterized by impaired compliance—information not reliably captured by imaging or endoscopy alone.

## 5 Implications for Therapeutic Endoscopy

### 5.1 Clinical Implications of a Biomechanics‐Informed Framework

Although most FLIP‐guided dilation strategies currently rely on diameter and distensibility measurements rather than on formal compliance calculations, emerging data suggest that incorporating biomechanical assessment may enhance interpretation of dilation response and help identify patients in whom further size escalation is unlikely to yield meaningful functional benefit.

- FLIP may be particularly useful when symptoms appear disproportionate to endoscopic findings or luminal diameter.
- Distensibility trends over time may be more informative than isolated measurements in growing children.
- Persistently impaired distensibility despite an adequate luminal diameter may suggest ongoing esophageal remodeling and warrants consideration when assessing potential response to additional dilation therapy.
- Normal endoscopic appearance does not exclude physiologically significant esophageal dysfunction.
- Discordance between symptoms, anatomy, and FLIP findings should prompt reassessment of underlying disease phenotype and alternative contributors to dysphagia.
- Etiology‐specific remodeling patterns likely influence reversibility and response to intervention.

### 5.2 Biomechanical‐Informed Dilation Strategies

Emerging pediatric studies suggest that FLIP can be used to guide esophageal dilation by providing objective, real‐time measurement of luminal diameter, reducing reliance on visual estimation or empiric size escalation [40, 41, 42, 43, 44, 45]. This capability directly addresses a long‐standing limitation in pediatric endoscopy, where endoscopic “eyeball” assessment of caliber is imprecise and poorly correlated with true luminal geometry, particularly in narrow or irregular strictures [2].

By shifting dilation from a predominantly size‐based intervention to a biomechanical one, FLIP reframes the procedural question from “how wide is the lumen” to “how does the esophageal wall respond to distension.”

Beyond improving diameter estimation, FLIP enables quantification of esophageal distensibility and, in research settings, compliance—metrics that reflect how the esophageal wall responds to applied distension rather than simply how wide the lumen appears. Currently, distensibility and diameter measurements represent the most clinically actionable FLIP parameters, whereas compliance‐based interpretation remains an evolving framework. Although standardized pediatric FLIP‐guided management algorithms remain unavailable, emerging evidence supports integration of FLIP‐derived biomechanical assessment into therapeutic decision‐making.

At present, the most actionable role of compliance assessment is not to dictate absolute targets, but to inform whether further dilation is likely to yield meaningful functional benefit versus diminishing returns [6, 15].

Available data suggest that etiology‐specific remodeling patterns may offer a framework for interpretation. Caustic and button battery injuries often demonstrate persistently low compliance despite increases in luminal diameter, consistent with transmural fibrosis [8, 9, 25], whereas many postsurgical anastomotic strictures exhibit focal stiffness with potential for gradual improvement in compliance over serial interventions [33, 34]. In contrast, vascular compression syndromes may show preserved intrinsic compliance with externally constrained luminal opening, supporting either conservative management or surgical referral depending on the presence of physiologically significant obstruction [37, 38]. Within this framework, FLIP‐derived measurements, including distensibility index, maximal diameter at standardized balloon fill volumes, and compliance across graded distension steps, may help determine whether further dilation is likely to be beneficial, rather than simply whether a target diameter has been achieved.

For example, persistently low compliance despite adequate diameter may signal incomplete remodeling and increased risk for symptom recurrence, prompting earlier reassessment of management strategy rather than repeated empiric escalation of dilation size. In select cases, temporary stent placement may provide sustained luminal expansion when repeated dilation fails. However, complications such as migration and the overall risk–benefit consideration must be weighed, and evidence that stenting favorably modifies underlying remodeling or improves compliance remains limited [6, 33, 34, 45]. Functional assessment may help identify patients most likely to benefit from this approach and guide timing of stent removal, though prospective data are needed. Collectively, these concepts support integration of FLIP‐derived biomechanical assessment into pediatric esophageal management.

## 6 Proposed Clinical Framework for Integrating FLIP Into Pediatric Esophageal Management

Although pediatric‐specific thresholds and standardized treatment algorithms remain incompletely defined, current evidence and cumulative clinical experience support several practical principles for integrating FLIP into pediatric esophageal evaluation and therapeutic decision‐making. Importantly, FLIP should be viewed as an adjunctive physiologic tool that complements, rather than replaces, symptom assessment, endoscopy, histology, radiographic imaging, and other esophageal physiologic testing [6, 15].

### 6.1 Which Patients May Benefit Most From FLIP Assessment?

At present, the most clinically useful applications of FLIP in pediatric practice include the following:

- Dysphagia or feeding dysfunction disproportionate to endoscopic findings
- Persistent symptoms despite apparently “adequate” luminal diameter after dilation
- Recurrent or refractory esophageal strictures requiring serial interventions
- Evaluation of post‐fundoplication symptoms, including suspected wrap dysfunction or impaired gastroesophageal junction opening
- Suspected fibrostenotic remodeling in eosinophilic esophagitis
- Evaluation of postsurgical narrowing, particularly after esophageal atresia repair
- Assessment of physiologic significance in vascular compression syndromes
- Real‐time intraprocedural assessment during dilation

In these settings, FLIP may provide physiologic information not captured by static anatomic assessment alone and may help contextualize discordance between symptoms, endoscopic appearance, and luminal caliber [6, 40, 42].

### 6.2 Which FLIP Metrics Currently Matter Most Clinically?

At present, the most clinically actionable FLIP parameters in pediatric practice are luminal diameter at standardized fill volumes and distensibility index, as these measurements are currently the best studied and most reproducible across disease states [6, 7, 16]. Longitudinal changes in these measurements over time may be particularly informative in children undergoing growth and repeated intervention.

In contrast, formal compliance calculations, contractile response phenotyping, and composite physiomechanical classification systems remain emerging investigational frameworks with promising but incompletely validated pediatric applications [5, 11, 22]. Accordingly, compliance should presently be interpreted primarily as a complementary conceptual framework that may help explain treatment response and symptom persistence rather than as a standalone determinant of management.

### 6.3 Interpreting Discordance Between Symptoms, Anatomy, and FLIP Findings

One of the most clinically valuable aspects of FLIP may be its ability to identify discordance between static anatomy and functional esophageal behavior (Table 1). For example, some children demonstrate relatively preserved luminal diameter but persistently impaired distensibility, suggesting ongoing biomechanical remodeling despite apparently adequate anatomic caliber [1, 3, 10]. Conversely, other patients tolerate relatively narrow lumens with preserved distensibility and minimal symptoms, indicating a functionally adaptable segment rather than a fixed obstructive phenotype.

**TABLE 1:** Practical interpretation of FLIP findings and potential management implications in pediatric esophageal disease.

Clinical scenario	Possible interpretation	Potential clinical implication
Narrow lumen with preserved distensibility	Functionally adaptable narrowing	Conservative management or cautious dilation escalation may be reasonable
Adequate diameter but persistently poor distensibility	Ongoing fibrosis/remodeling with persistently impaired biomechanics	Reassess anti‐inflammatory therapy, procedural strategy, or multidisciplinary management
Persistent symptoms with normal endoscopy but abnormal FLIP	Physiologic obstruction not captured by static anatomy	Consider functional narrowing or remodeling despite normal visual appearance
Persistent symptoms despite normalized FLIP metrics	Symptoms may not be driven primarily by biomechanical obstruction	Evaluate for dysmotility, visceral hypersensitivity, feeding dysfunction, or extraesophageal causes
Improved diameter without meaningful change in distensibility	Mechanical expansion without restoration of distensibility or compliance	Additional dilation alone may yield diminishing functional benefit
Preserved intrinsic distensibility with focal external narrowing	Extrinsic compression rather than intrinsic fibrosis	May support observation or targeted surgical evaluation depending on symptoms
Improved distensibility over serial evaluations	Favorable remodeling trajectory	Supports continued conservative or interval‐based management

Abbreviation: FLIP, functional lumen imaging probe.

Similarly, children with persistent dysphagia despite normal endoscopic findings may demonstrate physiologically significant narrowing or abnormal opening dynamics during FLIP assessment, whereas persistent symptoms despite normalized FLIP metrics should prompt consideration of alternative contributors such as esophageal dysmotility, visceral hypersensitivity, behavioral feeding dysfunction, or extraesophageal pathology [4, 11]. These discordant patterns highlight that symptoms, diameter, histology, and biomechanics represent related but distinct dimensions of esophageal disease severity.

### 6.4 How FLIP May Influence Dilation Strategy

Current pediatric dilation practice remains largely diameter‐based and symptom‐guided. However, FLIP introduces the possibility of function‐informed dilation strategies that incorporate biomechanical response in addition to static caliber [6, 43]. In practice, improvement in luminal opening and distensibility following dilation may support continued interval‐based management and observation. In contrast, persistently poor distensibility despite repeated size escalation may suggest diminishing functional benefit from increasingly aggressive dilation alone and should prompt reassessment of the broader management strategy. Failure of distensibility to improve despite serial diameter escalation may indicate that additional dilation alone is unlikely to provide durable symptomatic benefit.

Importantly, the goal of FLIP‐guided assessment is not necessarily to define a universal “target” diameter or distensibility threshold, but rather to help determine whether further mechanical intervention is likely to yield meaningful physiologic and symptomatic improvement. Within this framework, persistently abnormal biomechanical measurements despite apparently adequate caliber may support earlier optimization of anti‐inflammatory therapy, reconsideration of procedural intervals, multidisciplinary review, temporary stent placement in select cases, or surgical consultation depending on the underlying disease process [33, 45].

### 6.5 Developmental Considerations in Pediatric FLIP Interpretation

Interpretation of pediatric FLIP measurements requires careful consideration of developmental variability. Unlike adults, children undergo continuous changes in esophageal length, luminal caliber, wall composition, and swallowing behavior throughout growth and adolescence. Consequently, direct application of fixed adult distensibility thresholds to children is inappropriate and risks substantial misclassification [1, 17].

Developmental scaling may prove particularly important in younger children, where shorter esophageal length, smaller baseline caliber, sedation‐related physiologic effects, and feeding‐development variability can substantially influence FLIP measurements and contractile patterns. Accordingly, serial within‐patient trends over time may ultimately prove more clinically informative than isolated absolute measurements. This longitudinal, developmentally informed approach aligns with the broader concept that pediatric esophageal remodeling represents a dynamic biomechanical process evolving across years of growth, inflammation, healing, and repeated intervention rather than a static anatomic endpoint.

## 7 Future Directions

The expanding use of FLIP in pediatric esophageal disease has advanced the field beyond reliance on visually estimated diameter toward objective measurement of luminal geometry and biomechanical behavior. Although emerging studies demonstrate the feasibility of FLIP‐guided, diameter‐informed dilation, a key next step is establishing pediatric normative values and age‐adjusted reference standards for distensibility and compliance metrics. Another important priority is determining how compliance and distensibility metrics should be operationalized to guide therapeutic decision‐making. Prospective multicenter studies are needed to determine how changes in compliance relate to symptom response, durability of dilation, and risk of recurrence across different etiologies of esophageal narrowing. Longitudinal assessment will be particularly important in pediatric patients, in whom esophageal remodeling occurs over years of growth and repeated interventions. Developing pediatric‐specific frameworks that integrate diameter, compliance, and clinical context may ultimately allow dilation strategies to move beyond empiric size targets toward functionally informed endpoints, improving consistency of care and alignment with meaningful outcomes such as feeding tolerance, growth, and quality of life.

## 8 Conclusion

Pediatric esophageal disease cannot be fully understood through static anatomic measures alone. Esophageal biomechanics, including distensibility and compliance, provide complementary physiologic information that may better contextualize disease severity, remodeling, treatment response, and symptom discordance across heterogeneous pediatric esophageal disorders. Although distensibility remains the most clinically utilized FLIP metric, FLIP provides information beyond luminal diameter alone and may offer additional insight into esophageal function and remodeling. As pediatric experience continues to grow, integrating functional assessment with traditional anatomic evaluation may improve our understanding of stricture severity and help guide individualized management strategies.

## Funding

The authors have nothing to report.

## Conflicts of Interest

Brett J. Hoskins serves as a consultant for Medtronic plc, 3‐D Matrix Ltd., and Mirum Pharmaceuticals Inc. Kenneth Ng serves on the speaker's bureau for Abbott Laboratories and presents on FLIP‐related topics; he has no relationship with Medtronic plc. Michael A. Manfredi serves as a consultant for EvoEndo Inc.

## Data Availability

Data sharing not applicable to this article as no datasets were generated or analysed during the current study.
